# Development and validation of a community acquired sepsis-worsening score in the adult emergency department: a prospective cohort: the CASC score

**DOI:** 10.1186/s12873-024-01021-x

**Published:** 2024-06-20

**Authors:** François Saget, Adel Maamar, Maxime Esvan, Arnaud Gacouin, Jacques Bouget, Vincent Levrel, Jean-Marc Tadié, Louis Soulat, Paul Georges Reuter, Nicolas Peschanski, Bruno Laviolle

**Affiliations:** 1 Univ Rennes, CHU Rennes, service SAMU 35 / SMUR / Urgences Adultes, Rennes, F-35000 France; 2 Univ Rennes, CHU Rennes, Inserm, CIC, Centre d’investigation Clinique de Rennes (CIC1414), Service de Pharmacologie Clinique, Rennes, F-35000 France; 3 Service de Maladies Infectieuses et Réanimation Médicale, Häpital Pontchaillou, Université de Rennes 1, 2, rue Henri Le Guilloux, 35033 Rennes cedex 9, Rennes, France

**Keywords:** Sepsis, Septic shock, Emergency department, Prediction score

## Abstract

**Background:**

Sepsis is a leading cause of death and serious illness that requires early recognition and therapeutic management to improve survival. The quick-SOFA score helps in its recognition, but its diagnostic performance is insufficient. To develop a score that can rapidly identify a community acquired septic situation at risk of clinical complications in patients consulting the emergency department (ED).

**Methods:**

We conducted a monocentric, prospective cohort study in the emergency department of a university hospital between March 2016 and August 2018 (NCT03280992). All patients admitted to the emergency department for a suspicion of a community-acquired infection were included. Predictor variables of progression to septic shock or death within the first 90 days were selected using backward stepwise multivariable logistic regression to develop a clinical score. Receiver operating characteristic (ROC) curves were constructed to determine the discriminating power of the area under the curve (AUC). We also determined the threshold of our score that optimized the performance required for a sepsis-worsening score. We have compared our score with the NEWS-2 and qSOFA scores.

**Results:**

Among the 21,826 patients admitted to the ED, 796 patients were suspected of having community-acquired infection and 461 met the sepsis criteria; therefore, these patients were included in the analysis. The median [interquartile range] age was 72 [54–84] years, 248 (54%) were males, and 244 (53%) had respiratory symptoms. The clinical score ranged from 0 to 90 and included 8 variables with an area under the ROC curve of 0.85 (confidence interval [CI] 95% 0.81–0.89). A cut-off of 26 yields a sensitivity of 88% (CI 95% 0.79–0.93), a specificity of 62% (CI 95% 57–67), and a negative predictive value of 95% (CI 95% 91–97). The area under the ROC curve for our score was 0.85 (95% CI, 0.81–0.89) versus 0.73 (95% CI, 0.68–0.78) for qSOFA and 0.66 (95% CI, 0.60–0.72) for NEWS-2.

**Conclusions:**

Our study provides an accurate clinical score for identifying septic patients consulting the ED early at risk of worsening disease. This score could be implemented at admission.

**Supplementary Information:**

The online version contains supplementary material available at 10.1186/s12873-024-01021-x.

## Introduction

Sepsis is one of the leading causes of death and serious illness worldwide. After a long period of increasing incidence, sepsis is now actually decreasing, but mortality remains high [1]. It is defined as “life-threatening organ dysfunction caused by a dysregulated host response to infection“ [2]. Its early recognition and treatment are key parameters for improving overall survival, with an estimated in-hospital mortality over 27% [3]. In fact, the World Health Organization (WHO) made this a global health priority in 2017 to encourage research to improve the prevention, diagnosis and management of sepsis [4]. Early identification of patients suspected of having community-acquired sepsis who are at risk of worsening is therefore essential to improve these outcomes.

New definitions of sepsis and septic shock were published in 2016, abandoning the Systemic Inflammatory Response Syndrome (SIRS) criteria in favor of the Sequential Organ Failure Assessment (SOFA) score [2]. This score was validated in critically ill patients hospitalized in intensive care units (ICUs) and requires biological variables that are not rapidly available when patients arrive in the emergency department. Due to the difficulty of using this score outside the ICU, the Sepsis-3 working group proposed a simplified score, the quick SOFA (qSOFA), based on three clinical variables without any laboratory tests, and can be performed quickly and easily in the ward and emergency departments [2]. Since the creation of this score, many studies have questioned its performance. This score has an acceptable specificity but lacks sensitivity to predict mortality in in Emergency Department [5–9]. This performance may have deleterious consequences in the management of emergencies, leading to a delay in the implementation of therapies and consequently, an impact on morbidity and mortality [10, 11]. The latest recommendations of the Surviving Sepsis Campaign advise against its use as the sole screening tool for sepsis and septic shock [12].

Thus, unlike other critical illnesses, such as stroke, which have the National Institute of Health Stroke Scale (NOS) clinical score is used to assess patient prognosis and estimate severity [13], there is currently no reproducible and reliable consensus prognostic tool for sepsis in the emergency department.

Therefore, the aim of our study is to developed a prognostic clinical score that can rapidly identify a community acquired septic situation at risk of aggravation in patients consulting the emergency department.

## Methods

### Design and setting

The Community Acquired Sepsis Cohort (CASC) was a prospective, monocentric cohort study in the adult emergency department of Rennes University Hospital (NCT03280992). We included patients who visited our emergency department from March 2016 to August 2018 for a suspected community-acquired infection. The study was approved by the Rennes University Hospital’s ethical committee (no.16.15) and written informed consent was obtained from all subjects participating in the study. We followed the Transparent Reporting of a Multivariable Prediction Model for Individual Prognosis or Diagnosis (TRIPOD) reporting guidelines [14].

### Selection of participants, data collection and endpoints

The CASC cohort included patients ≥ 18 years hospitalized in the emergency department with a clinical suspicion of infection diagnosed by emergency physicians. In our study, we selected patients from the CASC cohort for whom infection was either proven, compatible on the basis of the clinical context or seen by radiological findings. Two experts (FS and JB) reviewed all the data and determined whether the acute presentation at the emergency department was related to infection by analyzing the following parameters: (i) positive culture of blood samples, respiratory samples, cytobacterial examination of urine, lumbar puncture, ascites puncture, and joint puncture; we also considered positive tests, such as specific urinary antigen test for *Legionella pneumophilae* or *Streptococcus pneumoniae*, polymerase chain reaction (PCR) for *Mycoplasma pneumoniae*, *Chlamydophila pneumoniae* and *Chlamydia psittaci*, and reverse transcriptase PCR for respiratory viruses; (ii) clinical context; and (iii) radiological findings. Moreover, the two experts assessed whether the infection was community acquired by analyzing if the patients were hospitalized in the ward or ICU within the three previous months.

We excluded patients who provided written opposition to the collection of their data, pregnant women and adults under legal protection (safeguard of justice, curatorship, guardianship) or deprived of freedom, with septic shock at admission, patients who were transferred from a different hospital prior to their admission with sepsis, and if the microbiological samples were collected before or after the ED stay to avoid including patients who did not meet the inclusion criteria simultaneously.

For each patient, clinical (including vital signs, medical history and treatments), and demographic (including place of residence) data were collected at admission and during emergency room management. The initial symptoms and medical management including the use of crystalloids or the need for oxygenation and ventilation support (nasal cannula, oxygen mask, non-invasive ventilation, mechanical ventilation) were recorded.

Sepsis and septic shock were defined according to the Sepsis-3 Definition [2]. Finally, the vital status was collected at 90 days, by telephone contact if necessary. If the patient did not respond, the attending physician was contacted as well as the town hall in order to have the exact date of death.

The primary endpoint was worsening of sepsis, defined as a composite endpoint of progression to septic shock or death within the first 90 days.

### Statistical analysis

Statistical analyses were performed using R 4.1.3 (R Foundation for Statistical Computing, Vienna, Austria), and *p*-values of less than 0.05 were considered significant.

Normally distributed continuous variables are presented as the means (standard deviations [SDs]), whereas non-normally distributed data are presented as medians (Interquartile ranges [IQRs]). The characteristics of patients who developed worsening of sepsis and those who did not were compared using Student’s t test or the Mann-Whitney test when appropriate for continuous variables, and the χ2 test or Fisher’s exact test when appropriate for categorical variables. Quantitative variables were recoded into categorical variables using the locally estimated scatterplot smoothing (LOESS) regression method [15] according to the points of inflection of the curve into two or three classes. Missing data were handled using multiple imputation [16]. Missing data at random were assumed. A total of 5 imputed datasets were generated, using 10 iterations. The accuracy and acceptability of the imputed data were evaluated with distribution plots.

To identify risk factors independently associated with worsening of sepsis, variables achieving a *p*-value < 0.1 between the two groups were entered into a backward stepwise logistic regression model. The results are expressed as odds ratios (ORs) with 95% confidence intervals (CIs). (Supplementary Table 1).

As previously described [17], the number of points assigned to each variable from the multivariable model corresponds to the value of the β coefficient of the model multiplied by 10 and rounded to the nearest integer. The score was then calculated for each patient, with mean scores for each group. A ROC curve was produced for each score and thresholds were defined to maximize sensitivity and specificity.

Predictive performance was expressed as discrimination (area under curve [AUC]) and calibration using the Hosmer-Lemeshow goodness-of-fit test, absence of collinearity between variables, and standardized deviance residual analysis. We also calculated the sensitivity, specificity, positive predictive value (PPV), and negative predictive value (NPV). The developed model was internally validated using bootstrap resampling [18]. Optimism-corrected performance was calculated as follows: optimism-corrected performance = apparent performance–optimism. We compared the results of our score with the qSOFA [2] and NEWS-2 [19]. Finally, we performed a sensitivity analysis of the application of the score on the complete cases.

## Results

### Sample characteristics

**Fig. 1:**
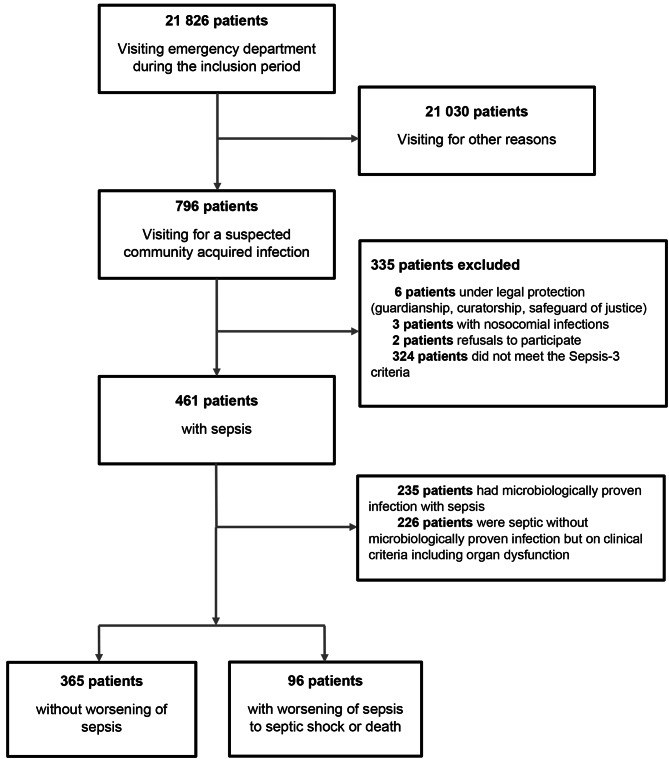
Flow diagram of study

During the study period, 21,826 patients consulted the emergency department of the University Hospital of Rennes (15,344 between 02/03/2016 and 16/06/2016, 6482 from 16/07/2018 and 27/08/2018), 796 of whom were included in our analysis. Among these patients with suspected community acquired infection, 461 met the inclusion criteria of our study with a sepsis. Among the 461 septic patients, 96 (21%) progressed to septic shock or death at D90. (Fig. 1).

Population was composed by 54% male with a median age of 72 [54–84] years. The place of residence prior admission to the emergency room was mostly home and 83 (18%) patients were discharged from a retirement home or a long-term care unit. Septic patients presenting to the emergency department had mainly respiratory symptoms (53%). The characteristics of these patients compared to those who did not progress to septic shock or death are presented in Table 1.

**Table 1:** Baseline clinical characteristics of study participants

Characteristics	Occurrence of septic shock or death at Day 90		*p*-value
	No (*N* = 365)	Yes (*N* = 96)	
Age, median [IQR], y	70 [48–82]	81.5 [67.75- 89]	*P* < .001
**Sex, No. (%)**			
Female	181 (50)	32 (33)	0.006
Male	184 (50)	64 (67)	
**Place of residence, No. (%)**			
Home	318 (87)	60 (63)	*P* < .001
Nursing home or long-term care unit	47 (13)	36 (37)	
**Physiological parameters**			
Systolic blood pressure, mean (SD), mmHg	134 (27)	120 (29)	*P* < .001
Diastolic blood pressure, mean (SD), mmHg	77 (16)	72 (17)	0.008
Mean blood pressure, mean (SD), mmHg	96 (20)	89 (21)	0.001
Heart rate, mean (SD), bpm	100 (20)	104 (22)	0.138
Temperature, median [IQR], °C	38.2 [37.3–38.9]	37.7 [36.8–38.6]	0.001
Respiratory rate, median [IQR], breaths/min	26 [20–32]	28 [24–33]	0.024
Glasgow coma scale, median [IQR],	15 [15–15]	15 [14–15]	*P* < .001
**Medical history**			
Active cancer, No. (%)	54 (15)	31 (32)	*P* < .001
Organ transplant, No. (%)	6 (2)	1 (1)	*P* > .99
Chronic inflammatory disease under immunosuppressant, No. (%)	11 (3)	5 (5)	0.344
Chronic viral infection treated, No. (%)	8 (2)	3 (3)	0.705
Hypertension, No. (%)	146 (40)	46 (48)	0.165
Diabetes, No. (%)	61 (17)	18 (19)	0.649
Dyslipidemia, No. (%)	81 (22)	25 (26.0)	0.417
Active smoking, No. (%)	44 (12)	10 (10)	0.725
Alcoholism, No. (%)	21 (6)	9 ( 9)	0.242
Heart failure, No. (%)	29 (8)	12 (13)	0.163
Coronary heart disease, No. (%)	39 (11)	9 ( 9)	0.852
Arterial disease, No. (%)	36 (10)	15 (16)	0.142
Cerebrovascular accident, No. (%)	37 (10)	15 (16)	0.147
Heart valve surgery, No. (%)	9 (3)	3 ( 3)	0.720
Chronic obstructive pulmonary disease, No. (%)	49 (13)	15 (16)	0.619
Chronic liver failure, No. (%)	4 (1)	2 (2)	0.609
Chronic renal failure, No. (%)	17 (5)	13 (14)	0.004
Disturbance of the known cognitive functions, No. (%)	31 (9)	31 (32)	*P* < .001
Splenectomy, No. (%)	3 (1)	1 (1)	*P* > .99
Beta-blocker therapy, No. (%)	72 (20)	15 (16)	0.463
ACE blockers or, ARB, No. (%)	74 (20)	17 (18)	0.666
**Symptoms on admission**			
Respiratory, No. (%)	175 (48)	69 (72)	*P* < .001
Abdominal, No. (%)	97 (27)	17 (18)	0.097
Genital or urinary, No. (%)	81 (22)	16 (17)	0.298
Cutaneous, No. (%)	41 (11)	6 (6)	0.213
Articular, No. (%)	21 (6)	3 (3)	0.439
Neuromeningeal, No. (%)	50 (14)	11 (12)	0.684
**Management**			
Room air ventilation, No. (%)	216 (59)	18 (19)	*P* < .001
Oxygenation and/or ventilation support, No. (%)	149 (41)	78 (81)	*P* < .001
Use of crystalloids, No. (%)	42 (12)	29 (30)	*P* < .001

Data are median [interquartile range] or mean (standard deviation) for continuous variables and number (%) for categorical variables

Definition of abbreviations: ACE, angiotensin-converting enzyme; ARB, angiotensin receptor blockers

### Clinical model development and validation

The final multivariable model retained eight predictors to establish the clinical score: sex, place of residence, Glasgow coma score, presence of cancer, use of crystalloids, oxygenation or ventilation support, presence of known cognitive impairment, and temperature. The regression coefficients and odds ratios of the final model are shown in Supplementary Table 2.

The prognostic performance of the clinical model is presented in Supplementary Table 3. The AUROC of the clinical model for predicting sepsis worsening was 0.85 (95% CI, 0.81–0.89) (Supplementary Fig. 1). The clinical model had a sensitivity of 0.81 (95% CI, 0.76–0.91), a specificity of 0.75 (95% CI, 0.70–0.79), and a negative predictive value was 0.94 (95% CI, 0.90–0.96) for predicting sepsis worsening. The Hosmer-Lemeshow test showed a *p*-value of 0.23 indicating a good calibration. The optimism corrected performance found by the bootstrap method (1000 iterations) was 0.84 indicating acceptable discrimination for internal validation.

### Clinical score

**Fig. 2:**
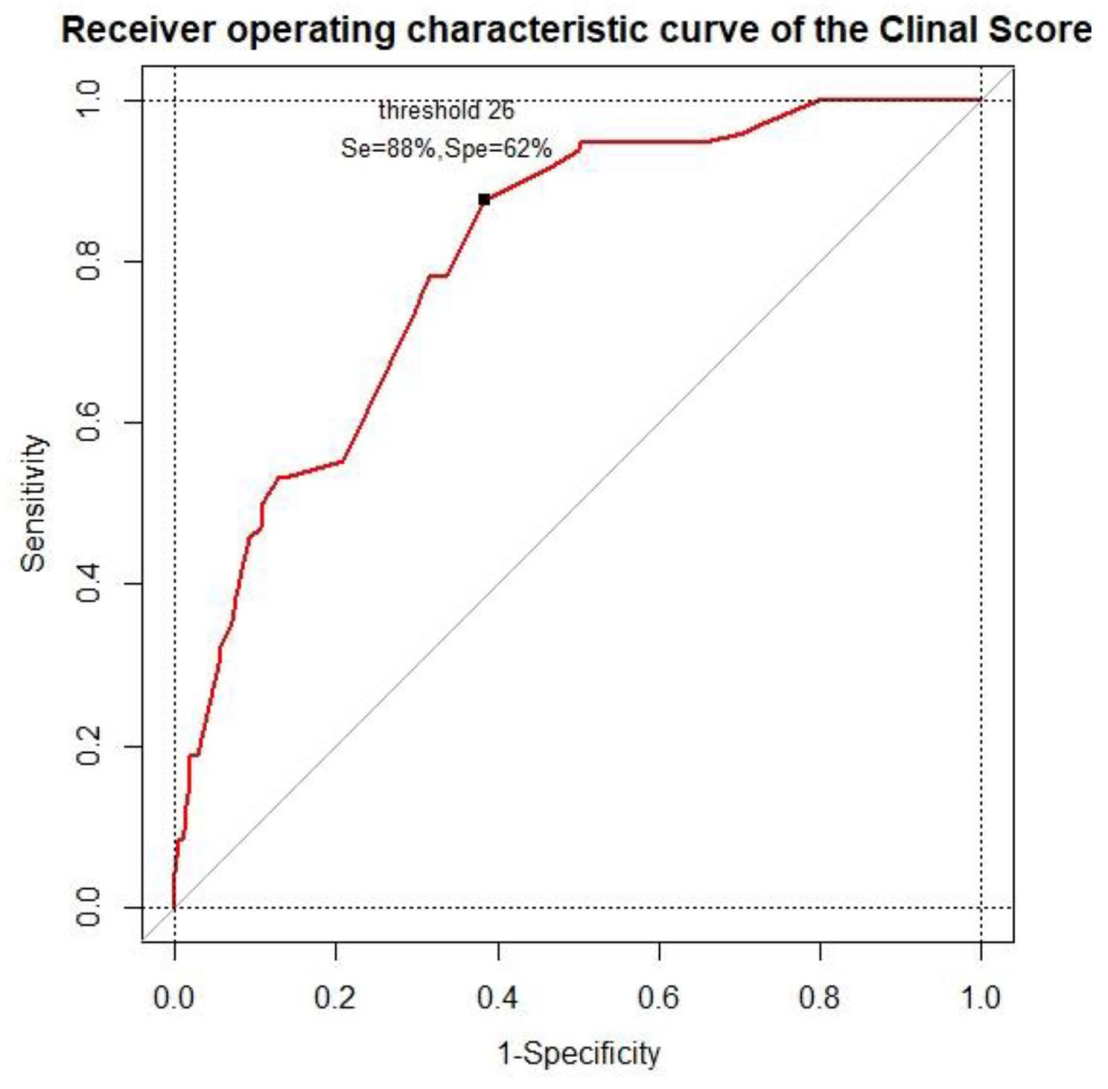
Clinical score receiver operating characteristic curve for predicting sepsis worsening

Table 2 shows the respective points attributed to each category of the clinical variable used to calculate the score. The points assigned to each variable vary from 0 to 16. Thus, the clinical score ranges from 0 to 90 (a higher score reflects a more severe prognosis). The median score was 24 [10–34] the group without worsening sepsis and 41 [32–50] in the group with worsening sepsis. A cut-off value of 26 was identified from the ROC curve as the best compromise between sensitivity (0.88, 95% CI 0.79–0.93) and specificity (0.62, 95% CI 0.57–0.67), while maintaining an excellent negative predictive value (0.95, 95% CI 0.91–0.97). (Fig. 2). The area under the ROC curve for CASC score was 0.85 (95% CI, 0.81–0.89) versus 0.73 (95% CI, 0.68–0.78) for qSOFA and 0.66 (95% CI, 0.60–0.72) for NEWS-2. We obtained a sensitivity for the qSOFA of 0.33 (95% CI, 0.24–0.43), and 0.49 (95% CI, 0.39–0.59) for the NEWS-2. (Supplementary Fig. 2).

**Table 2:** Clinical score to predict worsening of sepsis to septic shock or death at Day 90

Variables	Points					
	0	8	9	10	15	16
Sex	Female			Male		
Place of residence	Home	Nursing home				
Temperature	≥ 38 °C		< 38 °C			
Glasgow Score	Normal	< 15				
Active cancer	No				Yes	
Cognitive impairment	No				Yes	
Oxygenation and/or ventilation support	Ambiant air					Yes
Use of crystalloids fluids	No	Yes				

### Sensitivity analysis

A sensitivity analysis was performed on 407 complete cases. Eighty-six of these patients presented septic shock or death at Day 90. Using our clinical score on complete cases found 76 patients were considered true positives and 196 patients were considered as true negatives. The performance of the score in the sensitivity analysis is shown in Table 3.

**Table 3:** Diagnostic performance of the clinical score with threshold of 26 for prediction of sepsis worsening

For Prediction of worsening sepsis	Whole population	Sensitivity analysis population
Sensitivity % (95% CI)	0.88 (0.79–0.93)	0.88 (0.80–0.94)
Specificity % (95% CI)	0.62 (0.57–0.67)	0.61 (0.55–0.66)
Predictive value (95% CI)		
Positive	0.38 (0.31–0.44)	0.38 (0.31–0.45)
Negative	0.95 (0.91–0.97)	0.95 (0.91–0.98)
Likelihood ratio (95% CI)		
Positive	2.30 (1.98–2.67)	2.27 (1.94–2.66)
Negative	0.20 (0.12–0.34)	0.19 (0.11–0.34)

Definition of abbreviations: CI, confidence interval

## Discussion

In this study, we developed a new and easy to use clinical prediction score that presented a high accuracy to early identify community acquired septic patients at risk of deterioration into septic shock or death at 90 days. Its high negative predictive value is important for diseases such as sepsis where the risk of being undetected can lead to serious consequences. Indeed, delays in recognition of sepsis and administration of antibiotics are both associated with increased hospital mortality [11], especially after three hours of delay in the administration of antibiotics [20]. Consequently, our score could be used at the time of admission, using only clinical features, and might allow simple and rapid identification of patients at risk of deterioration.

While many severity scores currently in use are designed for critically ill patients and focus on predicting mortality, our score was specifically developed as a prediction tool [17, 21]. It presented a high sensitivity (88%) and negative predictive value (95%) to identify septic patients admitted to the emergency department at risk of worsening, superior to the qSOFA and NEWS-2 scores in our study which had sensitivities of 0.33 (95% CI, 0.24–0.43), and 0.49 (95% CI, 0.39–0.59) respectively, and negative predictive values of 0.84 (95% CI, 0.80–0.88) and 0.85 (95% CI, 0.82–0.89). These patients can subsequently be rapidly diagnosed and treated accordingly. Moreover, our score was created from an emergency population on routinely clinical data collected in the first hours of admission in an emergency department. Consequently, our score is not designed to replace the SOFA score, but could be complementary.

Interestingly, we found that fever was not associated with possible degradation. This may seem surprising in a septic context but could be explained by several points. First, 60% of adult septic patients are elderly, and thus are undergoing immunosenescence, which can lead to a decrease in cytokine production, alterations in the function and expression of Toll Like Receptors, and thymic involution [22]. This immunosenescence could explain why in 30–50% of cases, the elderly septic population does not present with fever [23]. Second, the fever of septic patients in the emergency room at admission may have been masked by the use of antipyretics. As fever helps in the recognition of sepsis and its early management, its absence probably delayed the recognition and, consequently, treatment initiation management explaining the severity of these patients.

Blood pressure did not appear to be a predictor of our score, which may seem surprising given the findings in the literature. Nevertheless, we assume that the assessment of hemodynamics and therefore blood pressure is assessed by the use of crystalloids. Elements of our score are found in several other scores. For example, the presence of cognitive impairment is found in the qSOFA [2], the NEWS-2 [19] and the MEDS score [24] and the place of residence is included in the MEDS score. In contrast, we believe that two variables are more comprehensive in our score: the need for ventilatory support and the use of crystalloids. Indeed, they are found to some extent in the MEDS score with tachypnea or hypoxia, in the NEWS-2 with oxygen administration, assessment of saturation and blood pressure, and finally in the qSOFA with respiratory rate and blood pressure. As far as temperature is concerned, we explained in the discussion why being apyretic can be a real danger in the emergency department which could lead to a delayed management and underecognition. We believe that this is an important point in our study, particularly in the geriatric population [22].

One strength of our score is its simplicity. This ease of application could allow to extend its use to the regulation of the Emergency Medical Services and thus trigger an alert signal that could reduce the delay in the administration of antibiotics [25]. The main limits our study was its monocentric design and no external validation cohort was used. Nevertheless, it was decided a priori to construct the score on the whole population and not to separate the sample into a derivation and a validation cohort. We validated our score using the Boostrap resampling method, and we found with 1000 iterations a corrected area under the curve close to our initial result. Second, as we aimed to develop a clinical score that can be used by the reception team, we excluded biological parameters and all features that are not routinely collected early after the admission. Therefore, we cannot rule out that using biological features could have enhanced the accuracy of our score. Another limitation is the use of cognitive impairment in our score rather than the Glasgow score. These two variables were statistically dependent in our analysis and are strongly clinically correlated, as the presence of a severe cognitive dysfunction impairs the Glasgow score. Thus, for our analysis, we selected the one that made the best contribution to our multivariable model.

## Conclusion

This study presents a simple and accurate clinical score that can rapidly identify community acquired septic patients at risk of deterioration among patients consulting the emergency department. Our score has to be validated in the future in sepsis case in general.

### Electronic supplementary material

Below is the link to the electronic supplementary material.

Supplementary Material 1

Supplementary Material 2

Supplementary Material 3

Supplementary Material 4

Supplementary Material 5

## Data Availability

The data that support the findings of this study are available from the corresponding author, FS, upon reasonable request.
