# Supplementary material for: Development and validation of a community acquired sepsis-worsening score in the adult emergency department: a prospective cohort: the CASC score
Source: BMC Emerg Med. 2024 Jun 20;24:102. doi: 10.1186/s12873-024-01021-x (PMC11188267; doi:10.1186/s12873-024-01021-x)

**Supplementary Figure 1.** Clinical model receiver operating characteristic curve for predicting sepsis worsening


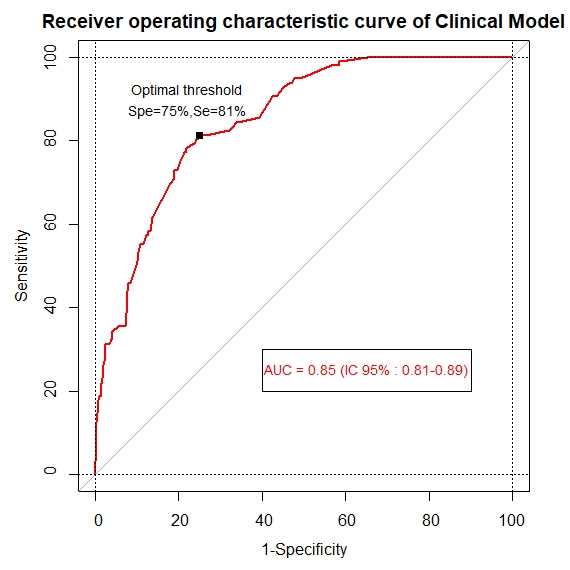

Supplement: Supplementary file 1 — Supplementary Material 1 [file 12873_2024_1021_MOESM1_ESM.docx]
