# Supplementary material for: Development and validation of a community acquired sepsis-worsening score in the adult emergency department: a prospective cohort: the CASC score
Source: BMC Emerg Med. 2024 Jun 20;24:102. doi: 10.1186/s12873-024-01021-x (PMC11188267; doi:10.1186/s12873-024-01021-x)
Supplement: Supplementary file 2 — Supplementary Material 2 [file 12873_2024_1021_MOESM2_ESM.docx]

**Supplementary Figure 2.** ROC curve comparing the CASC score with the NEWS 2 and qSOFA in predicting worsening sepsis**
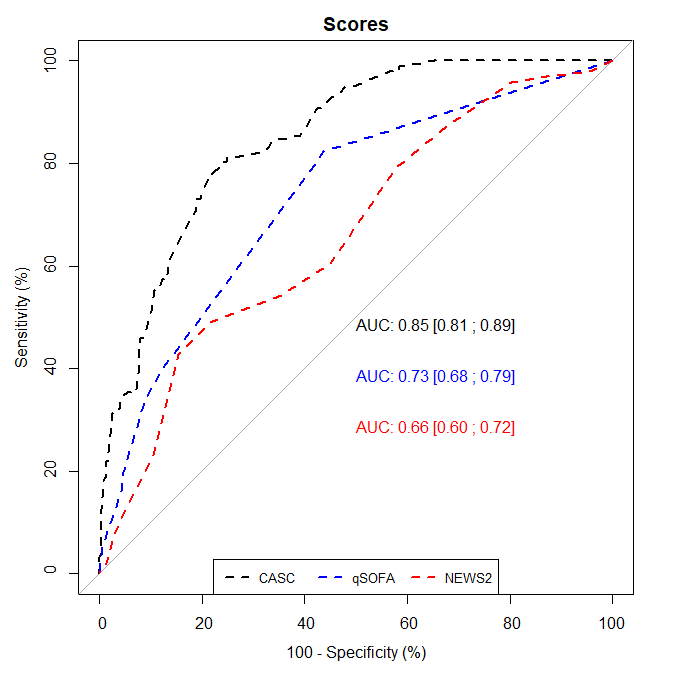
**
