# Supplementary material for: Development and validation of a community acquired sepsis-worsening score in the adult emergency department: a prospective cohort: the CASC score
Source: BMC Emerg Med. 2024 Jun 20;24:102. doi: 10.1186/s12873-024-01021-x (PMC11188267; doi:10.1186/s12873-024-01021-x)
Supplement: Supplementary file 3 — Supplementary Material 3 [file 12873_2024_1021_MOESM3_ESM.docx]

**Supplementary Table 1.** Univariate analysis

| **Univariate analysis** | | | |
| --- | --- | --- | --- |
| **Variables** | **OR** | **IC 95%** | **p-value** |
| Sex Male | 1.97 | 1.24 - 3.18 | <0.001 |
| Age (years) | 1.04 | 1.03-1.06 | <0.001 |
| Home | 0.25 | 0.15 - 0.41 | <0.001 |
| Nursing home or long-term care unit | 4.06 | 2.42 - 6.79 | <0.001 |
| Systolic blood pressure (mmHg) | 1.02 | 1.01 - 1.03 | <0.001 |
| Diastolic blood pressure (mmHg) | 1.02 | 1.01 - 1.04 | 0.008 |
| Mean blood pressure (mmHg) | 1.02 | 1.01 - 1.04 | 0.001 |
| Heart rate (bpm) | 1 | 1.00 - 1.02 | 0.138 |
| Temperature (°C) | 0.74 | 0.61 - 0.88 | 0.001 |
| Respiratory rate (bpm) | 1.07 | 1.04 - 1.10 | <0.001 |
| Glasgow coma scale | 0.67 | 0.53 - 0.81 | <0.001 |
| Active cancer | 2.75 | 1.63 - 4.59 | <0.001 |
| Organ transplant | 0.63 | 0.03 - 3.75 | 0.670 |
| Chronic inflammatory disease under immunosuppressant | 1.77 | 0.55 - 4.99 | 0.301 |
| Chronic viral infection treated | 1.44 | 0.31 - 5.09 | 0.596 |
| Hypertension | 1.38 | 0.88 - 2.17 | 0.162 |
| Diabetes | 1.15 | 0.63 - 2.02 | 0.638 |
| Dyslipidemia | 1.23 | 0.73 - 2.05 | 0.426 |
| Active smoking | 0.85 | 0.39 - 1.69 | 0.657 |
| Alcoholism | 1.69 | 0.72 - 3.72 | 0.205 |
| Insuffisance cardiaque | 1.66 | 0.78 - 3.31 | 0.167 |
| Heart failure | 0.86 | 0.38 - 1.78 | 0.709 |
| Coronary heart disease | 1.69 | 0.86 - 3.19 | 0.112 |
| Cerebrovascular accident | 1.64 | 0.84 - 3.08 | 0.133 |
| Heart valve surgery | 1.28 | 0.28 - 4.37 | 0.719 |
| Chronic obstructive pulmonary disease | 1.19 | 0.62 - 2.19 | 0.580 |
| Chronic liver failure | 1.92 | 0.26 - 9.99 | 0.455 |
| Chronic renal failure | 3.21 | 1.47 - 6.84 | <0.001 |
| Disturbance of the known cognitive functions | 5.14 | 2.92 - 9.07 | <0.001 |
| Splenectomy | 1.27 | 0.06 - 10.04 | 0.837 |
| Symptoms Respiratory | 2.77 | 1.72 - 4.59 | <0.001 |
| Symptoms Abdominal | 0.59 | 0.33 - 1.03 | 0.075 |
| Symptoms Genital or urinary | 0.7 | 0.38 - 1.24 | 0.239 |
| Symptoms Cutaneous | 0.53 | 0.20 - 1.19 | 0.157 |
| Symptoms Articular | 0.53 | 0.12 - 1.58 | 0.310 |
| Symptoms Neuromeningeal | 0.82 | 0.39 - 1.58 | 0.565 |
| Room air ventilation | 0.16 | 0.09 - 0.27 | <0.001 |
| Oxygenation and/or ventilation support | 6.25 | 3.67 - 11.17 | <0.001 |
| Use of crystalloids | 3.33 | 1.93 - 5.71 | <0.001 |
