# Supplementary material for: Development and validation of a community acquired sepsis-worsening score in the adult emergency department: a prospective cohort: the CASC score
Source: BMC Emerg Med. 2024 Jun 20;24:102. doi: 10.1186/s12873-024-01021-x (PMC11188267; doi:10.1186/s12873-024-01021-x)
Supplement: Supplementary file 4 — Supplementary Material 4 [file 12873_2024_1021_MOESM4_ESM.docx]

**Supplementary Table 2.** Variables associated with the occurrence of septic shock or death at Day 90 (multivariable model).

| **Effect** | **OR** | **CI 95%** | **p-value** |
| --- | --- | --- | --- |
| Male vs female | 2.63 | 1.48 - 4.84 | <0.01 |
| Nursing or long-term care unit vs home | 2.12 | 1.04 - 4.28 | 0.04 |
| Temperature <38 °C vs ≥ 38 °C | 2.36 | 1.36 - 4.17 | <0.01 |
| Glasgow coma scale < 15 vs normal | 2.18 | 0.99 - 4.75 | <0.05 |
| Active cancer Yes vs No | 4.40 | 2.30 - 8.54 | <0.001 |
| Cognitive disorders Yes vs No | 4.70 | 2.20 - 10.13 | <0.001 |
| Oxygenation and/or ventilation support vs Spontaneous room air ventilation | 4.97 | 2.70 - 9.55 | <0.001 |
| Use of crystalloids solution Yes vs No | 2.51 | 1.27 - 4.95 | <0.01 |

Definition of abbreviations: OR, odd-ratio; CI, confidence interval.
