# Supplementary material for: Development and validation of a community acquired sepsis-worsening score in the adult emergency department: a prospective cohort: the CASC score
Source: BMC Emerg Med. 2024 Jun 20;24:102. doi: 10.1186/s12873-024-01021-x (PMC11188267; doi:10.1186/s12873-024-01021-x)
Supplement: Supplementary file 5 — Supplementary Material 5 [file 12873_2024_1021_MOESM5_ESM.docx]

| **For prediction of sepsis worsening** | **Clinical model ^1^** |
| --- | --- |
| Sensitivity % (95% CI) | 0.81 (0.72 – 0.88) |
| Specificity % (95% CI) | 0.75 (0.70 – 0.79) |
| Predictive value (95% CI) |  |
| Positive | 0.46 (0.38 - 0.54) |
| Negative | 0.94 (0.90 - 0.96) |
| Likelihood ratio (95% CI) |  |
| Positive | 3.26 (2.66 - 3.99) |
| Negative | 0.25 (0.16 - 0.38) |
| AUROC (95% CI) | 0.85 (0.81 - 0.89) |

**Supplementary Table 3.** Diagnostic performances for the prediction of sepsis worsening

Definition of abbreviations: CI, confidence interval; AUROC, area under the receiver operating characteristic curve
